# Biologic Medications for Severe Asthma: Implications for Understanding Pathogenic Heterogeneity and Endotypes

**DOI:** 10.1146/annurev-med-070323-103158

**Published:** 2025-01-16

**Authors:** Richard P. Ramonell, Marc C. Gauthier, Anuradha Ray, Sally E. Wenzel

**Affiliations:** 1 Division of Pulmonary, Allergy, Critical Care, and Sleep Medicine, Department of Medicine, University of Pittsburgh, Pittsburgh, Pennsylvania, USA; 2 Asthma and Environmental Lung Health Institute at UPMC, University of Pittsburgh, Pittsburgh, Pennsylvania, USA; 3 Department of Environmental and Occupational Health, University of Pittsburgh, Pittsburgh, Pennsylvania, USA

**Keywords:** severe asthma, biologic therapy, molecular phenotypes, endotypes, eosinophils, cytokines

## Abstract

Asthma is a chronic inflammatory disease of the airways long known for phenotypic heterogeneity. Phenotyping studies in asthma have led to a better characterization of disease pathogenesis, yet further work is needed to pair available treatments with disease endotypes. In this review, the biology of targeted pathways is discussed along with the efficacy of biologic therapies targeting those pathways. Results of asthma clinical trials are included, as well as results of trials in related diseases. This review then analyzes how biologics help to inform the complex immunobiology of asthma and further guide their use while identifying areas for future research.

## INTRODUCTION

Asthma, which affects more than 300 million people worldwide, results in variable symptoms and airflow limitation (1). It is driven by a complex set of inflammatory processes characterized by clinical and pathophysiological heterogeneity (2). Efforts to understand disease pathogenesis on a molecular level have resulted in improved understanding of the mechanisms that underlie asthma (3). However, our understanding of these disease pathways and ability to tailor therapy based on them remains incomplete.

Biologic medications effectively eliminate signaling through specific immunological pathways. In asthma, they have offered fascinating insights into underlying mechanisms that drive aberrant physiology and, ultimately, asthma symptoms. However, despite their specificity and biomarker-driven use, there is considerable heterogeneity in treatment responses ranging from disease remission to nonresponse. Thus, biologic medications offer opportunities to refine our pathophysiological understanding of asthma heterogeneity. In this review, we discuss how the differing efficacy of biologic medications for asthma, including differences between biologic classes and clinical responses among different subgroups, iteratively informs our understanding of the underlying pathobiologies and potentially improves our treatment choices. For more detailed discussions about the efficacy and safety of biologic medications in asthma, we refer the reader to multiple comprehensive reviews (3–5).

## THE CONCEPTS OF PHENOTYPING AND ENDOTYPING

**Figure 1:**
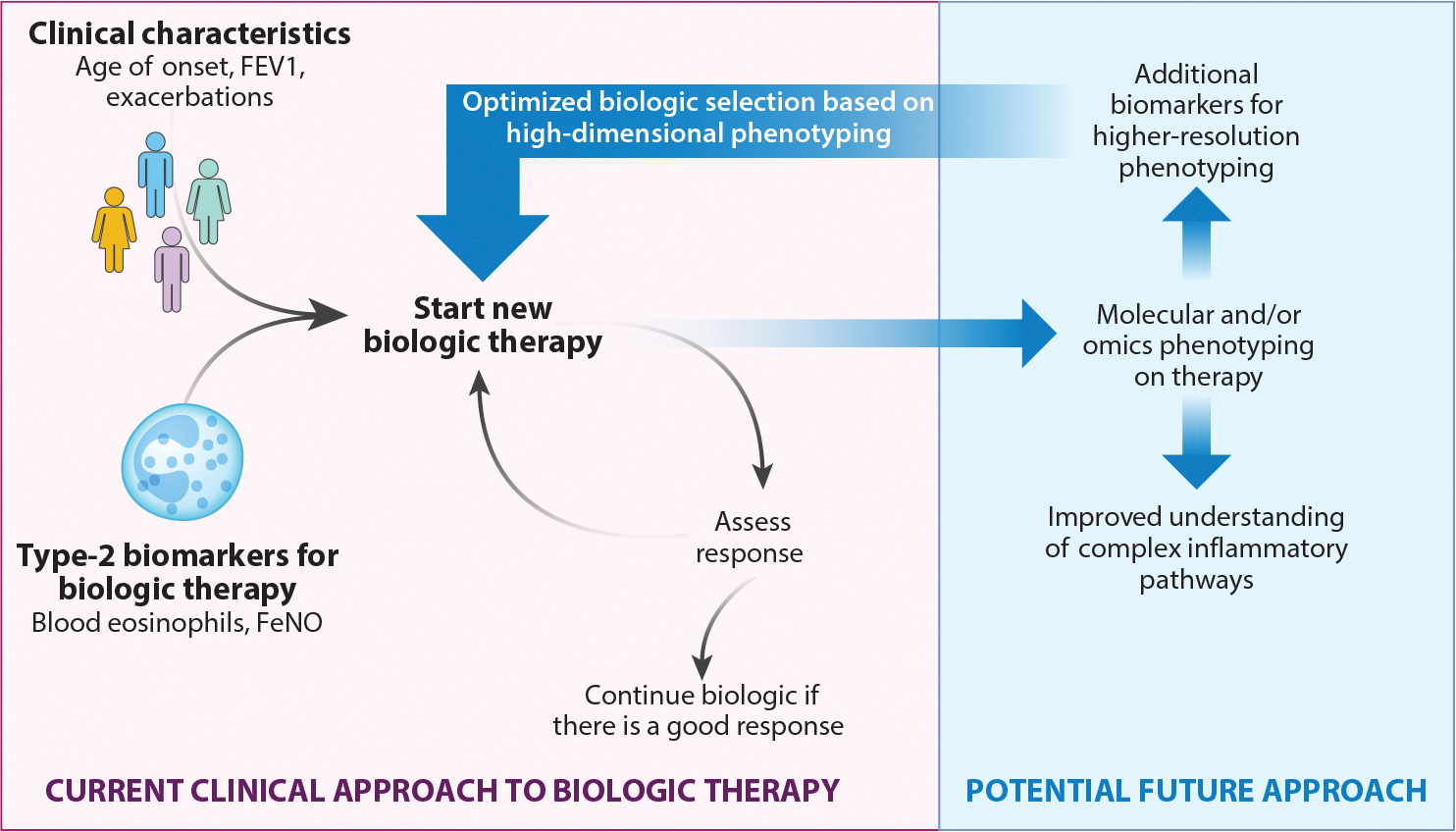
Tailoring biologic therapy to molecular phenotypes. Molecular phenotypes are derived from clinical (*top left*) and molecular characteristics (*bottom left*) in patients with asthma. In carefully selected patients who meet criteria for biologic medications, a biologic therapy should be initiated. Current practice includes assessment of the treatment response, yet future studies should utilize omics approaches to both improve endotyping and develop novel biomarkers to identify disease phenotypes. Abbreviations: FeNO, fraction of exhaled nitric oxide; FEV1, forced expiratory volume in 1 s.

Whereas a phenotype is defined as the measurable physical properties of an organism that result from interactions between the environment and genetic factors (6), a molecular phenotype integrates both clinical and molecular characteristics, including disease biomarkers and more broad omics approaches (7) (Figure 1). A molecular phenotype differs from an endotype in that an endotype is defined as a grouping of patients in which a pathobiological pathway has been identified that, when blocked or augmented, critically determines the activity or progression of the disease (7). As such, endotyping ultimately allows precision medicine, delivering targeted therapies to those most likely to respond (8).

## THE EVOLUTION OF PHENOTYPING IN ASTHMA

Asthma has long been recognized as a heterogeneous disease with differing presentations and courses (9). Early phenotyping efforts described patients with early-onset disease, sensitization to aeroallergens, and concomitant allergic rhinitis as having extrinsic asthma; these patients differed from individuals with later-onset disease more often associated with infections and nasal polyposis, often without clinical evidence of atopy (intrinsic asthma) (10). This framework was widely accepted until the 1990s when biopsy studies reported consistent inflammatory features across asthma patients (11–13) including elevations in newly identified type-2 (T2) cytokines like interleukin (IL)-4, IL-5, and IL-13 (14, 15). Additionally, broad anti-inflammatory medications like oral corticosteroids (OCSs) and, later, inhaled corticosteroids (ICSs) were also shown to be generally effective in most patients regardless of intrinsic or extrinsic phenotype. These studies contrasted with studies from the 1950s (recapitulated in the 2000s) showing that the efficacy of corticosteroids (CSs) was tied to the presence of sputum eosinophils and, later, T2 status itself (16, 17). In parallel, inflammatory studies of lung samples identified subsets of asthma with elevated tissue or sputum eosinophils, often in association with distinct clinical features (18–21). Most recently, small studies of anti-IL-5 confirmed efficacy only in the presence of eosinophilic inflammation. Combined, these concepts promoted the phenotypes of eosinophilic and/or T2-high asthma and T2-low asthma that are widely used today. T2-high patients are now generally defined by an absolute eosinophil count (AEC) ≥150 cells/μL or a fraction of exhaled nitric oxide (FeNO) ≥25 ppb.

**Figure 2:**
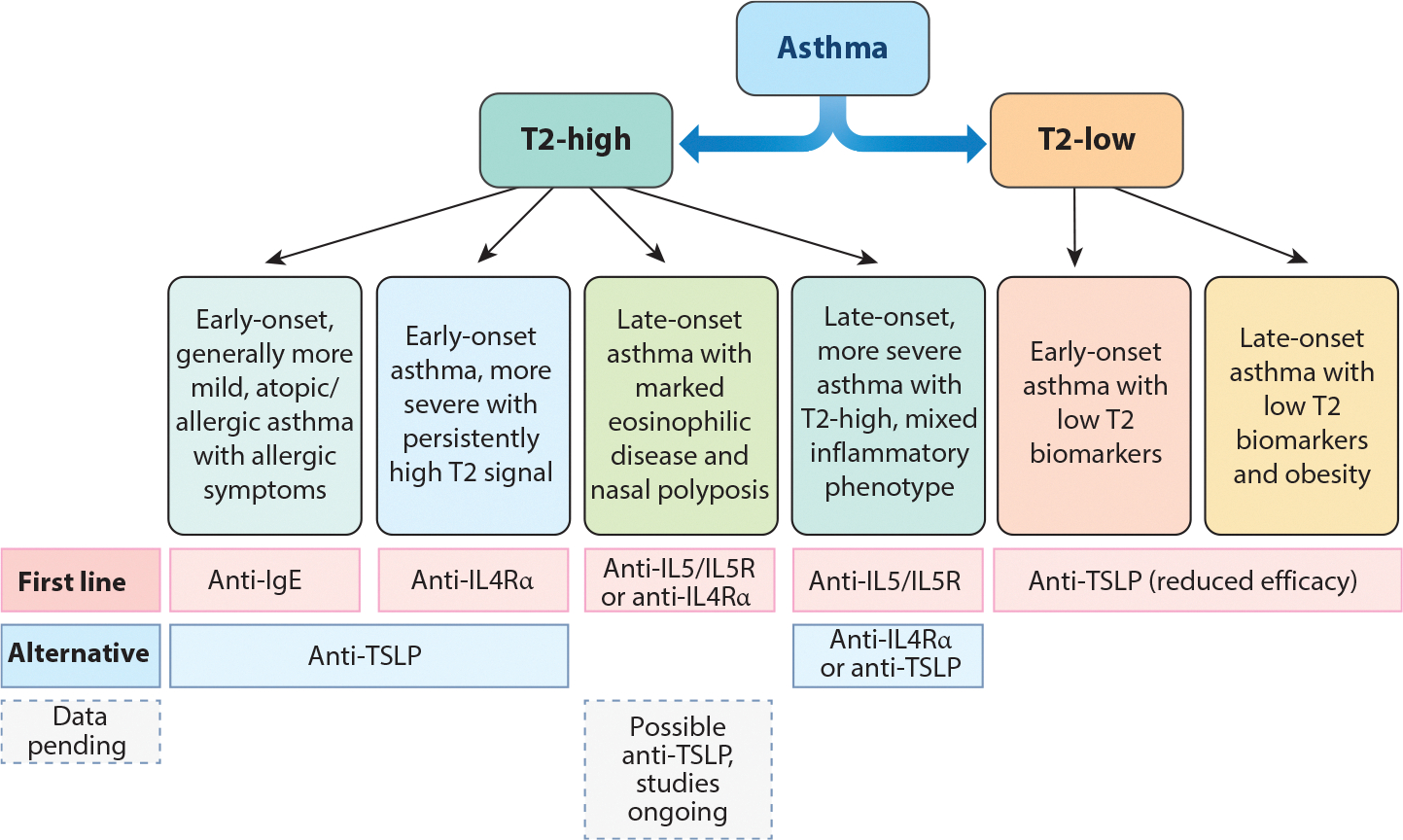
Diagram of broadly recognized T2-high and T2-low molecular phenotypes and subphenotypes. Phenotyping analyses that include molecular data and clinical metadata reveal largely consistent phenotypes, with differing potential responses to biologic therapies. These phenotypes are initially stratified by the presence (T2-high) or absence (T2-low) of T2 inflammation as measured by AEC and FeNO. Molecular phenotypes within T2-high or T2-low patients are clustered based on both clinical characteristics (age of asthma onset, atopy, nasal polyps, and obesity) and their relation to readily available T2 biomarkers. Suggested first-line (*top row*) and alternative (*second row*) biologics are listed below each molecular phenotype based on data from clinical trials and inference from basic and translational studies. Abbreviations: AEC, absolute eosinophil count; FeNO, fraction of exhaled nitric oxide; IgE, immunoglobulin E; IL, interleukin; R, receptor; T2, type 2; TSLP, thymic stromal lymphopoietin.

While this eosinophilic/T2-high phenotyping strategy now dominates clinical practice, there is recognition of heterogeneity within these groups (9). Attempts have been made to more deeply phenotype using large cohorts, such as the National Heart, Lung, and Blood Institute Severe Asthma Research Program (22, 23) and the European Unbiased Biomarkers for the Prediction of Respiratory Disease Outcomes study (24, 25), among others (26). Combining large clinical datasets with later molecular data from sputum, these studies identified variable numbers of clusters, but consistent phenotypes emerged including: (*a*) mild early-onset allergic asthma, (*b*) more severe early-onset T2-high asthma, (*c*) later-onset T2-high asthma with nasal polyps or chronic sinusitis, (*d*) later-onset T2-high asthma with mixed inflammatory phenotype, (*e*) early-onset T2-low asthma, and (*f*) later-onset T2-low asthma with obesity and minimal obstruction but significant symptoms (27) (Figure 2). These phenotypes can be further modified by smoking status and other environmental exposures (28, 29), which may augment late-onset T2-high disease or use of high-dose CSs and elicit a pauci-immune, more airway-centric disease (25, 26).

## BIOLOGIC MEDICATIONS AS A PLATFORM

Biologic medications are a class of genetically engineered humanized monoclonal immunoglobulin (Ig)G antibodies. In asthma, these medications are highly specific for their antigenic targets and generally have favorable safety profiles. Approved asthma biologic antibodies target critical points surrounding the T2 immune response including T2 cytokine signaling (IL-4, IL-5, and IL-13), downstream T2 response (IgE), and upstream T2 initiation [thymic stromal lymphopoietin (TSLP)]. In clinical trials, these biologics improved exacerbations, clinical symptoms, and airway obstruction as measured by forced expiratory volume in 1 s (FEV1) (4). Except for tezepelumab (anti-TSLP), these biologics are approved for asthma with specific biomarker elevations relevant to these pathways: sensitization to a perennial aeroallergen (anti-IgE), elevated AEC [anti-IL-5, anti-IL-5 receptor (IL-5R), anti-IL-4Rα], and increased FeNO (anti-IL-4Rα). These biomarkers largely fall into the existing clinical eosinophilic/T2-high paradigm, but significant heterogeneity exists within this dichotomy. This fact is reflected by variable biologic efficacy across asthmatic and extrapulmonary disease. Using more granular asthma clinical and molecular phenotypes could allow better targeting of existing biologics to the patients most likely to benefit from them, while the ability of biologics to effectively eliminate certain pathways could open new research insights into complex molecular pathways, hopefully resulting in more granular and specific biomarkers for precision asthma therapy.

## ANTI–IMMUNOGLOBULIN E (OMALIZUMAB)

### Pathway Biology: Immunoglobulin E and Allergic Inflammation

IgE is a class of antibody partly responsible for coordination of antiparasitic and, of more relevance in developed countries, allergic responses. IgE is produced by plasma cells and binds to the surface of cells expressing the high-affinity (FcεRI) or low-affinity (FcεRII) IgE receptor (30). In patients with asthma, IgE is primarily expressed bound to surface FcεRI on mast cells and basophils. When IgE binds to its target allergen, it cross-links with other cell-bound IgE molecules (and receptors) and initiates a cascade of events beginning with degranulation followed by lipid mediator generation and cytokine production (30).

### Clinical Efficacy

Omalizumab is a recombinant humanized IgG1κ monoclonal antibody that selectively binds to the constant region of the heavy chain of IgE, preventing its ability to complex with the IgE receptor (Table 1). It was the first biologic medication to gain US Food and Drug Administration (FDA) approval for the treatment of severe asthma. Anti-IgE attenuates the fall in FEV1 associated with both the early and late asthmatic responses to inhaled allergens to which the patient is sensitized, confirming the role of IgE (31, 32). A phase III study of patients with allergic asthma (defined as elevated serum IgE in the presence of at least one positive allergen-specific skin prick test) on medium-to-high-dose ICS showed a nearly 50% reduction in asthma exacerbations compared to placebo (0.28 exacerbations per patient over the 16-week steroid stable phase in the omalizumab group versus 0.54 in controls, *p* = 0.006) coupled with an ability to reduce ICS doses but few other clinical improvements (33). It is important to note that IgE levels (or allergen sensitivity) are not predictive of omalizumab responses, with a post hoc analysis of seven pooled trials confirming that IgE levels did not predict response (34). Further, the efficacy appears to be lower in patients with severe allergic asthma as currently defined (35), with asthma exacerbation rates reduced by only 25% (0.66 exacerbations per patient in the omalizumab group versus 0.88 in controls, *p* = 0.006) (36). Omalizumab is efficacious in children with severe asthma (by definition with early-onset asthma) as young as 6 years of age, reducing exacerbations by 38–43% (37, 38). Interestingly, omalizumab also reduced the known increase in return-to-school viral asthma exacerbations, suggesting that its efficacy extends beyond traditional IgE–allergen blockade (38).

### Efficacy in Special and Related Conditions

Subsequent studies of omalizumab demonstrated efficacy in chronic rhinosinusitis with nasal polyposis (CRSwNP) (39). However, the data supporting efficacy in atopic dermatitis are controversial, with a small but significant effect reported in children (40). In contrast, omalizumab in a single dose is highly efficacious in preventing symptoms of allergic rhinitis when an injection is given prior to the ragweed pollen season (41).

### Impact on Asthma Phenotyping

Clinical and molecular phenotyping studies identified an early-onset asthma phenotype that is strongly associated with atopy and allergy (42). This phenotype is broadly defined as asthma that begins in early childhood and is associated with allergen-specific IgE, often in association with other allergic conditions such as rhinitis or atopic dermatitis. There can be a vast range of severity, but typically patients respond well to ICS (42). This phenotype of patients, typically with more severe disease, was initially targeted for omalizumab therapy.

While omalizumab is a highly (perhaps the most) effective treatment for laboratory-induced asthmatic responses to allergens in mild asthma (31, 32), it is considerably less efficacious in adults meeting the current definition of chronic severe allergic asthma, with only an approximately 25% reduction in exacerbations (36). In fact, the presence of T2 biomarkers, more so than total IgE level, predicts treatment response to omalizumab in post hoc studies (43). One potential explanation for this finding lies in the differences in allergic asthma pathobiology between children and adults. Whereas chronic allergic asthma in adults is characterized by more complex airway biology, pediatric allergic asthma may be primarily a mast cell and/or basophil disorder reliant on cell surface–bound IgE.

Collectively, these findings suggest that blockade of allergen-induced asthmatic responses is clinically effective only in subsets of patients in whom mast cell and IgE-mediated immune effects drive disease. IgE-driven disease tends to be more prevalent in younger individuals with milder disease and perhaps with stronger allergic rhinitis symptoms. Future studies are required to determine the molecular pathways beyond IgE that may critically define a more severe allergic asthma endotype.

## ANTI-EOSINOPHIL THERAPY (ANTI–INTERLEUKIN 5/INTERLEUKIN 5 RECEPTOR)

### Pathway Biology: Eosinophils in Asthma

Eosinophils are granulocytes that participate in innate immune responses. In healthy humans, eosinophils are predominantly found in the skin and gastrointestinal tract, where they are poised to rapidly combat parasitic infections (44). However, patients with asthma have elevations in airway-tissue, sputum, and peripheral-blood eosinophils (45), where their numbers have been associated with disease severity and exacerbations (46, 47). Eosinophils exert their effects primarily through the release of granular inflammatory proteins and proteases, but they may also be capable of activating immune responses through antigen presentation (48, 49). Eosinophils canonically rely on IL-5 for bone marrow maturation, cell activation, and survival (50, 51), and therefore, both IL-5 and its receptor emerged as treatment targets in asthma.

### Clinical Efficacy

Biologic medications have been developed against the IL-5 molecule (mepolizumab and reslizumab) and the alpha subunit of the IL-5 receptor (IL-5Rα; benralizumab). Whereas reslizumab and mepolizumab prevent IL-5 signaling by binding and inactivating free IL-5, benralizumab binds IL-5Rα and induces IgG1-mediated antibody-dependent cellular cytotoxicity against eosinophils and basophils, effectively removing these cells from peripheral blood (Table 1) (52).

#### Mepolizumab and reslizumab.

Both mepolizumab and reslizumab target IL-5 but are on different antibody backbones: Mepolizumab is a humanized IgG1κ monoclonal antibody, whereas reslizumab is a humanized IgG4κ monoclonal antibody. Early studies of mepolizumab, unlike those with anti-IgE, demonstrated profound eosinophil depletion but little effect on airway hyperresponsiveness or obstruction following inhaled allergen challenge (53). A subsequent study of mepolizumab in moderate asthma patients not preselected for peripheral blood eosinophil level demonstrated only a 10% reduction in exacerbations (54). In landmark investigator-initiated studies that preselected patients for evidence of airway eosinophilia, mepolizumab reduced annualized asthma exacerbations compared to placebo and lowered oral CS doses in patients previously dependent on them (55). These findings have been replicated in larger, multicenter studies of both mepolizumab (56–59) and reslizumab (60, 61), with consistent reduction in asthma exacerbations by 40% to 50%. These studies determined that a blood eosinophil count of >300 cells/μL could be substituted for sputum eosinophilia for the prediction of efficacy.

#### Benralizumab.

Benralizumab is also a humanized IgG1κ monoclonal antibody. Yet a key difference is that benralizumab targets IL-5Rα and induces antibody-dependent cellular cytotoxicity resulting in eosinophil apoptosis (52, 62, 63). While this has been suggested to improve the ability to target tissue eosinophils, data to support this are modest, potentially due to the reported loss of the IL-5R on eosinophils in tissue (64). Like cytokine-targeted approaches, benralizumab reduced exacerbations in patients with uncontrolled eosinophilic asthma on medium to high dose ICS and long-acting β-agonist (LABA) with the greatest efficacy in individuals with an AEC of >300 cells/μL (62). Benralizumab decreased the annualized risk of asthma exacerbations by 36% to 45% in two phase III studies (62, 63), with modest improvement in FEV1. In contrast to omalizumab, benralizumab was not as effective in milder asthma, failing to improve FEV1 (65). Similar to mepolizumab, a recent study of benralizumab in an allergen challenge model was also negative (66), suggesting that eosinophils are not essential to allergic asthmatic responses. Mepolizumab has also been evaluated in severe asthmatic children aged 6 to 17 with an AEC of >150 cells/μL. Mepolizumab treatment reduced the annualized rate of asthma exacerbations compared to placebo by 28% without affecting FEV1 or symptom scores (67).

### Efficacy of Anti–Interleukin 5 in Special and Related Conditions

Both anti-IL-5 and anti-IL-5R biologics have shown efficacy in decreasing OCS doses in patients previously dependent on them. Mepolizumab decreased OCS doses by 50% compared to the placebo group while decreasing the annualized asthma exacerbation rate (1.44 versus 2.12, *p* = 0.04) (58). Benralizumab similarly decreased OCS doses in patients with severe eosinophilic asthma by 75%, compared with 25% reduction in the placebo group, while reducing exacerbations (68). Notably, patients in both studies had evidence of persistent eosinophilia even while taking OCS (prior to anti-IL-5/IL-5R treatment). While both mepolizumab and benralizumab are effective in eosinophilic asthma, only mepolizumab improves polyp size and nasal obstruction compared to placebo in patients with CRSwNP (69). Benralizumab was not effective (70).

Anti-eosinophil biologics are the only class with reported efficacy in eosinophilic granulomatosis with polyangiitis (EGPA). EGPA is a small-to-medium-vessel vasculitis that differs from other vasculitides because of its propensity to cause asthma and prominent peripheral eosinophilia (71). Mepolizumab was FDA approved for the treatment of EGPA after showing efficacy in a phase III trial (72). Benralizumab was found recently to be noninferior to mepolizumab in inducing remission in relapsing or refractory EGPA (73).

### Impact on Asthma Phenotyping

Use of anti-IL-5 pathway biologics has greatly contributed to our understanding of T2-high/eosinophilic phenotypes. Eosinophils have long been associated with allergen-induced airway responses (74), yet anti-IL-5/IL-5R agents are not effective in blocking responses to allergens (66), calling into question the role of eosinophils (and anti-IL-5) in early-onset, allergic asthma. Indeed, patients with late-onset asthma and nasal polyps (typically associated with late-onset asthma) are more likely to respond to these therapies than patients with early-onset asthma, as supported by a retrospective study examining pooled data from benralizumab trials (75) and a post hoc analysis of two phase III benralizumab trials (76). Benralizumab responses are enhanced in patients with older ages at diagnosis and nasal polyposis as well as more severe disease, including OCS use and low lung function. The more modest efficacy of mepolizumab in children with eosinophilic asthma is consistent with these response predictors in adults. They are also consistent with an increasing importance of eosinophils (as opposed to IgE) to disease as severity increases.

Interestingly, although patients with asthma and concomitant nasal polyps appear to respond particularly well to anti-eosinophil biologics, only mepolizumab is FDA approved for the treatment of CRSwNP (69, 70). These results suggest differences between upper and lower airway disease responses, as well as differences in anti-IL-5 versus anti-IL-5R approaches in patients with CRSwNP. Understanding these differences requires further investigation.

## ANTI–INTERLEUKIN 4 RECEPTOR α

### Pathway Biology: Type-2 Inflammation in Asthma

IL-4 and IL-13 are two prominent T2 cytokines in asthma (77–79). T2 inflammation was classically associated with adaptive immune responses activated through the transcription factor GATA3 (77) following T cell activation by antigen-presenting cells in the presence of IL-4 (79). Production of IL-4 then magnifies type 2 helper T cell (Th2) polarization while mediating B cell class switching to IgE (80). Th2 cells can also produce IL-5 and IL-13, contributing to additional effects (11, 12, 78). Additional sources of T2 inflammation include mast cells, which generate IL-4 and IL-13 (81), as well as type-2 innate lymphoid cells, which also produce similar T2 cytokines, most specifically IL-5 and IL-13 (82). Eosinophils (83) and airway stromal cells (84) have also been reported to produce IL-13.

Both IL-4 and IL-13 can contribute to many of the observed airway changes seen in asthma, including airway hyperreactivity, goblet cell hyperplasia, mucous production, smooth muscle proliferation, and subepithelial basement membrane thickening (85). IL-4 and IL-13 signal through a shared chain (IL-4Rα) (86). While only IL-4 can signal through IL-4Rα dimerized with the common γ chain, both IL-4 and IL-13 signal through IL-4Rα when dimerized with the IL-13Rα1 (86). The expression of this second heterodimer on a wide variety of cell types likely explains the broad activities of IL-13. As IL-4Rα is common to both receptors, it may explain some of the overlap between the effects of IL-4 and IL-13 (85).

### Clinical Efficacy of Dupilumab

The shared IL-4Rα subunit for IL-13 and IL-4, which signals via JAK–STAT, provides a target for more global T2 inflammatory suppression (86). Dupilumab is an IgG4κ monoclonal antibody against IL-4Rα that effectively blocks IL-4 and IL-13 signaling (Table 1) (87). Dupilumab significantly reduced exacerbations between 48% and 71% relative to placebo with dosing every 2 weeks across phase IIb and III studies (88, 89). Dupilumab also showed efficacy in children (ages 6 to 11) with a 59% reduction in exacerbations and a 5.2% increase in FEV1 predicted compared to placebo (90). Unlike treatment with anti-IL-5 modulators, IL-4Rα blockade consistently improved FEV1 by nearly 150 mL compared to placebo (89). Allergen challenge studies with dupilumab have not been performed, but studies of pitrakinra (a competitive inhibitor for IL-4Rα) showed significant efficacy in blocking both early and late asthmatic allergen responses, suggesting efficacy in blocking IL-4Rα in allergen-mediated disease and contrasting with IL-5 pathway blockade (91). Dupilumab efficacy improves with increasing T2 biomarker status as measured by AEC and FeNO (88, 89, 92). Patients with elevations in both had the highest risk of exacerbations and also the greatest improvement with dupilumab, further supporting this concept. FeNO, in addition to being a predictive biomarker for response to dupilumab, is a response biomarker for this treatment, as the degree of FeNO reduction correlates with improvement in FEV1 (89, 93).

While a discussion of adverse events is not included for the other biologics, dupilumab has a more unique side effect profile, which may be related to its biology and thus may contribute to understanding its mechanisms and even endotypes. Dupilumab use is associated with eosinophilia in clinical trials, which has been observed in 4.1% of patients with nearly a third of these patients reaching an AEC of >3,000 cells/μL. Of the participants, seven stopped therapy due to eosinophilia and four (0.6%) developed potentially related symptoms (89). Post-approval, case reports and series have described a rise in seronegative arthritis (94), psoriasis (95), and IL-17-mediated disease (96). However, the relation to these side effects is likely complex, as dupilumab was reported to be efficacious in alopecia areata (97) and to potentially contribute to worsening alopecia (98). Whether these effects are due to release of a T2 brake on T1 or T17 polarization remains to be determined.

### Efficacy of Anti–Interleukin 4 Receptor α in Special and Related Conditions

Dupilumab is effective in controlling atopic dermatitis (99), long associated with asthma, particularly in childhood. It is also efficacious in CRSwNP (100). Like blockade of IL-5 pathways, dupilumab treatment led to a 70% reduction in OCS dose compared to 42% in placebo (*p* < 0.001), even without selecting for patients with evidence of eosinophilic inflammation (92). This broad efficacy supports a role for dupilumab in patients with more severe and global T2 disease.

### Impact on Asthma Phenotyping

IL-4Rα therapy may be most effective in markedly T2-high patients (adult and pediatric) with elevated levels of both FeNO and eosinophils, especially those with comorbid T2 disease where dupilumab has shown clinical efficacy such as CRSwNP (101) or atopic dermatitis (102). Notably, IL-13 blockade with lebrikizumab (103) and tralokinumab (104) (anti-IL-13 antibodies) did not show efficacy in phase III trials, suggesting that blockade of both IL-4 and IL-13 is necessary. The hypereosinophilia observed in trials suggests caution in patients with a markedly high AEC, and use is generally discouraged for those with an AEC of >1,500 cells/μL. The emergence of T1- and T17-mediated diseases on dupilumab therapy suggests that patients with a more mixed inflammatory phenotype (105) or combined neutrophilic/eosinophilic asthma (106) should be monitored closely if a trial of dupilumab is attempted. It remains to be determined whether skewing of adaptive immunity to T1 or T17 following broad blockade of T2 inflammation occurs in certain individuals and drives these side effects.

## ANTI-ALARMIN THERAPY

### Pathway Biology

Alarmins, primarily IL-33 and TSLP, are a class of cytokines produced by airway epithelial and other cells that play important roles in initial host defense (107). IL-33 is a member of the IL-1 family and elicits Th2 responses via the receptor ST2 (IL-1 receptor like 1) (108). IL-33 is released from airway epithelium in response to allergen exposure and can trigger IL-13 release from mast cells (109). It may also be important in asthmatic responses to viruses, as its absence in an RSV infection model in mice blunted airway hyperreactivity (110). TSLP is produced in the lungs by a variety of cells, including macrophages, epithelial cells, submucosal cells, mast cells, and neutrophils (111). TSLP signals through a heterodimeric receptor composed of IL-7Rα and TSLP-γ (112). Both IL-33 and TSLP undergo cleavage, particularly by mast cell proteases, yielding many different protein forms with different reactivity (107).

### Clinical Efficacy of Tezepelumab

Tezepelumab is a human IgG2λ monoclonal antibody against TSLP that binds and prevents interaction with its receptor (Table 1). A phase IIb study showed a 71% reduction in annual exacerbation rate with tezepelumab compared to placebo (113). Similar to dupilumab, tezepelumab improved FEV1 by 110 mL compared to placebo. A subsequent pivotal trial showed similar improvements in annualized exacerbation rate (56% reduction) and FEV1 (130-mL improvement) compared to placebo (114). Unlike any other biologic, subgroup analyses stratified by T2 status showed improvements in exacerbation rates even in those with low AEC (<150 cells/μL) or FeNO (<25 ppb). However, the greatest improvements were still seen in patients with high blood eosinophils (>300 cells/μL) and/or FeNO (>50 ppb), showing that efficacy was still tied to T2 status. Notably, both FeNO and blood eosinophils decrease following treatment with tezepelumab. However, these data led to the approval of tezepelumab in severe asthma without biomarker qualifications.

### Efficacy of Anti–Thymic Stromal Lymphopoietin in Special and Related Conditions

Unlike anti-IL-5/IL-5R or anti-IL4R, tezepelumab was not effective in lowering OCS doses compared to placebo (115). This may have been influenced by a large OCS reduction in the placebo arm, and a subgroup of participants with T2-high status did show significant OCS reduction. Unlike dupilumab, tezepelumab was ineffective at controlling atopic dermatitis (116). It remains unclear whether tezepelumab is truly a biomarker-nonspecific severe asthma therapy. In fact, eosinophils were the only cell type that decreased in airway tissue following treatment with tezepelumab (117), supporting its T2-directed biology.

### Impact on Asthma Phenotyping

Tezepelumab is efficacious in T2-high asthma with some efficacy in patients with low levels of T2 biomarkers as well (although efficacy is more pronounced in patients with elevated T2 biomarkers). Whether this efficacy is related to residual T2 signal (not reflected in AEC or FeNO) or effects of alarmin inhibition on other pathways is not clear. Allergen challenge studies have shown an ability to blunt asthmatic early and late allergen responses (118), suggesting a benefit in environmental- or allergen-triggered disease. TSLP may be different from other alarmins, as anti-IL-33 therapy with itepekimab showed reductions in exacerbations but was inferior to dupilumab (positive control in the study), with combination therapy (itepekimab + dupilumab) faring worse than dupilumab alone (119). This suggests that alarmins and T2 cytokines are not necessarily interchangeable as treatment targets. It remains to be seen whether alarmin therapy will show a benefit in broader T2-high conditions, as seen with dupilumab, or whether its efficacy is more limited to the airways, given the initial data in atopic dermatitis. As such, its efficacy across differing asthma phenotypes is still being evaluated.

## CONCLUSIONS

The advent of biologic therapies has significantly improved treatment options for patients with severe asthma. While all currently approved biologics reduce exacerbations and are to some degree T2 targeted, they still display unique treatment profiles in terms of optimal patient age (or age of disease onset), degree of eosinophilia, presence of additional T2-mediated disease (atopic dermatitis, nasal polyposis, etc.), and in their ability to address lung function. Based on approaches combining biomarkers, biologics, and where possible, omic or pathobiological studies, we propose dupilumab as the first-line therapy targeted to adults and children with severe asthma with elevated FeNO and eosinophils, especially in cases with CRSwNP and no evidence of autoimmune disease (Figure 2). We alternatively propose the use of anti-eosinophil therapies for patients with consistently high eosinophils, often with very severe disease, including OCS dependency, typically of later onset. Omalizumab should be considered for adult and pediatric patients with moderately severe early-onset asthma and is perhaps best in those with a link between allergen exposure, symptoms, and elevated T2 (as opposed to allergic) biomarkers. We also propose tezepelumab as the first-line treatment in those without consistent elevation in T2 biomarkers, but further guidance in relation to tailoring biologics, particularly tezepelumab, to molecular phenotypes is needed.

Future prospective studies are needed to truly identify asthma endotypes in which a specific pathway substantially drives disease. Consideration should be given to the identification of super-responders, defined as patients who have a marked improvement in asthma control and lung function, a reduction or elimination of OCS, and a reduction or elimination of exacerbations following biologic initiation (120). Expansion of the use of biologic medications for asthma quickly revealed not only the existence of these individuals but also their prevalence (14–44% depending on the definition used) (121). While these super-responders to T2 biologics presumably have severe asthma as a result of a canonical T2-associated inflammatory pathway, the identification of additional factors that differentiate these super-responders from those with less responsive disease requires further investigation. Prospective, placebo-controlled studies investigating clinical and molecular characteristics of these patients are critical as we approach true endotyping in asthma.

While the likelihood of clinical response to biologics is partially informed by the degree of T2 status, it is not absolute, suggesting that better targeted biomarkers and phenotyping are also needed. In turn, appropriate studies of biologics may identify additional biomarkers that better predict efficacy. Matching clinical, molecular, and other omics data to specific biologic responses could help clarify the complexity of asthma pathobiology. However, despite these unanswered questions, the combination of targeted biologics with even the current biomarkers and associated phenotypic characteristics have greatly improved our ability to treat a variety of patients with severe asthma.

## Figures and Tables

**Table 1:** FDA-approved biologic medications for severe asthma

Biologic	Patient age	Phase IIb/III studies (reference)	Unique inclusion criteria	Relative reduction in annualized exacerbation rate (exacerbation/patient/year)^a^	Change in prebronchodilator FEV1^a^	Validated asthma symptom questionnaires^a^	Asthma control^a^
Omalizumab (anti-IgE)	Adult	Busse et al. (33) Solèr et al. (122) Hanania et al. (36)	+skin test to ≥ 1 perennial allergen IgE 30–700 IU/mL Total omalizumab calculated dose ≤700 mg	Stable ICS phase: 48–58% ICS reduction phase: 41–52%	+2% predicted	AQLQS: +0.29 (95% CI, 0.15–0.43)	SABA use: −0.27 puff/day (95% CI, −0.49 to −0.04)
	Pediatric	ICATA (38)		38% (95% CI, 18–58%)	No change	Reduction in asthma symptom days: 24.5%	Significant decrease in ICS and LABA use
Mepolizumab (anti-IL-5)	Adult	MENSA (57) MUSCA (59)	≥2 asthma exacerbations in the prior 12 months AEC ≥300 cells/μL in the prior 12 months and ≥ 150 cells/μL at screening	53–58%	+98 to 120 mL	SGRQ: −7.0 to −7.7	ACQ5: −0.40 to −0.44
	Pediatric	MUPPITS-2 (67)	AEC ≥150 cells/μL	27% (95% CI, 4–44%)	No difference	Patient rated effective: Drug: 84% Placebo: 89%	Physician rated effective: Drug: 66% Placebo: 71%
Reslizumab (anti-IL-5)	Adult	Study 1/2 (60) BREATH (61)	AEC ≥400 cells/μL ACQ7 ≥1.5	54% (95% CI, 42–67%)	+110 to 160 mL	AQLQ: +0.23 to 0.36	ACQ7: −0.25 to −0.32
Benralizumab (anti-IL-5R)	Adult	CALIMA (62) SIROCCO (63)	≥2 exacerbations in the prior 12 months ACQ6 ≥1.5	28–51% (95% CI, 5M6%) (in patients with AEC ≥300 cells/μL)	+116 to 169 mL (in patients with AEC ≥300 cells/μL)	AQLQ: +0.24 to +0.30	ACQ6: −0.25 to −0.22
Dupilumab	Adult	Wenzel et al. (88) Liberty Asthma Quest (89)	ACQ5 ≥1.5 ≥ 1 systemic CS treatment or hospital admission for asthma	66–81% (in patients with AEC ≥300 cells/μL)	+150 to 240 mL (in patients with AEC ≥300 cells/μL)	AQLQ: +0.24 to +0.30 (in patients with AEC ≥300 cells/μL)	ACQ6: −0.25 to −0.22 (in patients with AEC ≥300 cells/μL)
	Pediatric	VOYAGE (90)	≥ 1 severe exacerbation in the prior 12 months	59.3% (95% CI, 39.5–72.6%)	+5.2% predicted (95% CI, 2.1–8.3%) (predicted)	PAQLQ: +0.34 (95% CI, 0.16–0.52)	ACQ7: −0.28
Tezepelumab (anti-TSLP)	Adult	PATHWAY (113) NAVIGATOR (114)	≥2 severe exacerbations in the prior 12 months ACQ5 ≥1.5	56–71% (41% in patients with AEC ≥300 cells/μL)	+8.26% predicted (PATHWAY) +130 mL (NAVIGATOR) +70 mL (in patients with AEC ≥300 cells/μL)	AQLQ: +0.18 to +0.34 (+0.21 in patients with AEC ≥300 cells/μL)	ACQ6: −0.27 to −0.33 (−0.21 in patients with AEC ≥300 cells/μL)

a Relative to placebo, range of reported mean values in all phase IIb/III trials.

Abbreviations: ACQ, Asthma Control Questionnaire score (MCID −0.5); AEC, absolute eosinophil count in blood; AQLQ, Asthma Quality of Life Questionnaire score; CI, confidence interval; CS, corticosteroid; FDA, US Food and Drug Administration; FEV1, forced expiratory volume in 1 s; ICS, inhaled corticosteroid; Ig, immunoglobulin; IL, interleukin; LABA, long-acting β-agonist; MCID, minimal clinically important difference; PAQLQS, Pediatric AQLQ score; R, receptor; SABA, short-acting β-agonist; SGRQ, St. George Respiratory Questionnaire score (MCID −4); TSLP, thymic stromal lymphopoietin.
